# 

**Published:** 1889-04-13

**Authors:** 


					. flo. 133, Vol. YL-
April 13th, 1889.
t I /^egistered at the General Post Office as a Newspaper.]
T^Tir frill H ' MAHlflly MfL?, ill.; puyi ii'uu, i3u.
/ Ditto per Annum, 7s. 6d., post free.

				

